# A Non-Coronary, Peripheral Arterial Atherosclerotic Disease (Carotid, Renal, Lower Limb) in Elderly Patients—A Review: Part I—Epidemiology, Risk Factors, and Atherosclerosis-Related Diversities in Elderly Patients

**DOI:** 10.3390/jcm13051471

**Published:** 2024-03-03

**Authors:** Marcin Piechocki, Tadeusz Przewłocki, Piotr Pieniążek, Mariusz Trystuła, Jakub Podolec, Anna Kabłak-Ziembicka

**Affiliations:** 1 Department of Vascular and Endovascular Surgery, The St. John Paul II Hospital, Prądnicka 80, 31-202 Krakow, Poland; mpiech98@gmail.com (M.P.); tpkardio@kki.pl (P.P.); m.trystula@szpitaljp2.krakow.pl (M.T.); 2 Department of Cardiac and Vascular Diseases, Institute of Cardiology, Jagiellonian University Medical College, św. Anny 12, 31-007 Krakow, Poland; tadeuszprzewlocki@op.pl; 3 Department of Interventional Cardiology, The St. John Paul II Hospital, Prądnicka 80, 31-202 Krakow, Poland; jjpodolec@gmail.com; 4 Department of Interventional Cardiology, Institute of Cardiology, Jagiellonian University Medical College, św. Anny 12, 31-007 Krakow, Poland; 5 Noninvasive Cardiovascular Laboratory, The St. John Paul II Hospital, Prądnicka 80, 31-202 Krakow, Poland

**Keywords:** atherosclerosis, biomarkers, cardiovascular events, cardiovascular risk, carotid artery lesions, elderly patients, extra coronary arterial disease, prognostic factors, peripheral arterial disease, renal artery stenosis

## Abstract

Atherosclerosis is a generalized and progressive disease. Ageing is a key risk factor for atherosclerosis progression that is associated with the increased incidence of ischemic events in supplied organs, including stroke, coronary events, limb ischemia, or renal failure. Cardiovascular disease is the leading cause of death and major disability in adults ≥ 75 years of age. Atherosclerotic occlusive disease affects everyday activity and quality of life, and it is associated with reduced life expectancy. Although there is evidence on coronary artery disease management in the elderly, there is insufficient data on the management in older patients presented with atherosclerotic lesions outside the coronary territory. Despite this, trials and observational studies systematically exclude older patients, particularly those with severe comorbidities, physical or cognitive dysfunctions, frailty, or residence in a nursing home. This results in serious critical gaps in knowledge and a lack of guidance on the appropriate medical treatment and referral for endovascular or surgical interventions. Therefore, we attempted to gather data on the prevalence, risk factors, and management strategies in patients with extra-coronary atherosclerotic lesions.

## 1. Introduction

Atherosclerosis is a generalized and progressive disease [1,2]. Ageing is a key risk factor for atherosclerosis progression that is associated with increased incidence of ischemic events in supplied organs, including stroke, coronary events, limb ischemia, or renal failure [3,4]. Presently, nearly 10% of the European Union population is 75 years or older. Cardiovascular disease is the leading cause of death and major disability in elderly patients [5,6]. Atherosclerotic occlusive disease affects everyday activity and quality of life, and it is associated with reduced life expectancy [6,7]. Although there is much evidence on the coronary artery disease (CAD) management in the elderly, there is insufficient data on older patients’ management presented with atherosclerotic lesions outside coronary territory. Despite this, trials and observational studies systematically exclude older patients, particularly those with severe comorbidities, physical or cognitive dysfunctions, frailty, or residence in a nursing home [8,9]. This results in serious critical gaps in knowledge and a lack in the guidance on the appropriate medical treatment and referral for endovascular or surgical interventions.

Therefore, we attempted to gather data on the prevalence, risk factors, management strategies, and medical treatment of patients with extra-coronary atherosclerotic lesions, indicating where there is some evidence on the management in elderly patients and where there are gaps in the evidence-based medicine.

### 1.1. Arterial Ageing

#### 1.1.1. Atherosclerosis

Atherosclerosis is a chronic, inflammatory-thrombotic disease, and its progression usually depends on the age of the patient [1]. Its development is determined by both genetic and environmental factors, with the latter playing a fundamental role [10,11]. Atherogenic processes are already programmed in fetal life and initiated in the early years of an individual’s development [10,11,12,13]. Approximately 10% of males aged 40 years already have well-developed atherosclerotic plaques, and by the age of 60 years, atherosclerosis is diagnosed in more than half of them [14,15]. Environmental factors play an essential role in the rate of development of atherosclerotic lesions; therefore, effective cardiovascular prevention programs are crucial in the management of these diseases [16,17,18]. Despite the implementation of recommendations regarding diet and physical activity, as well as pharmacotherapy for the management of cardiovascular risk factors, atherosclerosis is a progressive disease in more than 80% of people [19,20,21]. Hence, age remains the main prognostic factor for the presence and progression of atherosclerosis.

Since atherogenic processes occur at different rates in in various individuals, it is worth remembering that vascular age (i.e., the actual condition of the vessels) prevails over chronological age. In approximately 35–40% of individuals, there is a discrepancy between chronological age and vascular age that can occur in either direction [22]. According to imaging studies, approximately one-fifth of individuals who are deemed to have a low cardiovascular risk based on the Framingham scale (less than 5% risk of death and cardiovascular events in a 10-year follow-up) have atherosclerotic plaques in their carotid arteries that are greater than 1.5 mm in thickness. This finding indicates that even a ‘healthy’ person may be at a higher risk of cardiovascular events [23]. Similarly, in patients over the age of 70 years, the expected common carotid intima–media complex thickness (CIMT) should not exceed 0.9 mm (i.e., the upper percentile for age and sex according to the results of epidemiological studies) [24,25]. Conversely, with a growing CIMT thickness, there is a growing number of arterial territories with advanced atherosclerosis lesions [26].

#### 1.1.2. Vascular Remodeling, Arterial Stiffness, and Compliance

Atherosclerosis is inextricably linked to vascular ageing, which involves the loss of elastic fibers and their replacement with collagen and calcium, leading to vessel stiffening [27]. The muscular walls of young arteries contain a large amount of elastin, which helps to regulate their tension and maintain proper vascular compliance in response to the shock wave of blood flowing from the left ventricle during each cardiac cycle. The arteries must absorb this impact by altering their diameter in systole and diastole to avoid being damaged, dissected, or ruptured.

With age, a considerable proportion of elastin fibers is lost and substituted with collagen. This phenomenon is unfavorable because it causes a reduction in arterial compliance. This decrease is linked to increased tension in the vascular wall, endothelial dysfunction, greater resistive index (RI) and pulsatile index (PI), and an increase in pulse wave velocity (PWV).

Additionally, increased vascular stiffness contributes to an increase in blood pressure (BP), which leads to further unfavorable remodeling of the arteries [28]. Both increased vascular stiffness and hypertension generate oxidative stress and chronic inflammation, which lead to the development and progression of atherosclerosis. This phenomenon is particularly noticeable in the bifurcations of arteries due to turbulent blood flow and unfavorable distribution of tension forces in the vessel wall. The bifurcation of the common carotid artery into the internal and external carotid arteries meets the above conditions and is also easily accessible for examination. Therefore, it is an ideal location to detect the first signs of atherosclerosis.

Each of these elements: decreased arterial compliance, increased vessel stiffness measured using PWV or RI, endothelial dysfunction, switching of smooth muscle cells to proliferation, and the appearance of atherosclerotic plaques and calcifications are prognostic factors for increased mortality due to stroke, myocardial infarction (MI), or circulatory failure, as well as all-cause mortality [29,30]. It is assumed that PWV values above 9 m/s and RI above 0.7 indicate a two- to threefold increase in cardiovascular risk [30]. However, in people over the age of 65 years, the cut-off point for PWV may be higher. In a Japanese study involving 530 patients having a mean age of 76 ± 5.6 years, with PWV values above 19 m/s, it was shown that individuals had a tenfold greater risk of cardiovascular death (CVD) (HR 10.01, 95% CI 1.21–82.49) at the 3 year follow-up [31].

Taken together, it is clear that age is an unmodifiable risk factor for vascular stiffness and atherosclerosis, independent of other atherogenic factors [32,33].

## 2. Methodology

A narrative review of the epidemiology, prevalence, and risk factors of extracardiac atherosclerotic occlusive disease in elderly was conducted. Public PubMed databases were searched using the following query: (‘peripheral arterial disease’ OR ‘PAD’ OR ‘renovascular disease’ OR ‘RVD’ OR ‘renal artery stenosis’ OR ‘RAS’ OR ‘carotid artery disease’ OR ‘ICAS’) AND (‘cardiovascular risk factors’) AND (‘epidemiology’ OR ‘incidence’ OR ‘prevalence’) AND (‘elderly’ OR ‘genarians’). The occurrence of the above terms in the title or abstract was checked for all articles written in English without specifying a timeframe and filtering by article type. A study was included in this narrative review if it fulfilled the following predefined inclusion criteria: (1) involved a population with at least one of the following conditions: PAD, ICAS, RAS; (2) described the epidemiology or risk factors; (3) included data on the elderly.

Search results yielded 477 articles. Abstracts of these studies were reviewed to include those that fulfill the inclusion criteria. Case reports were excluded. When duplicates were identified, the most recent study was included unless the earlier version reported more relevant outcomes. The above criteria were met by the 156 studies included in the review.

## 3. The Faces of Atherosclerosis

### 3.1. Atherogenesis in Different Locations

The development of atherosclerotic plaques and their characteristics change over time as well as with the advancing age of the patient [34,35,36]. Atherosclerotic plaques undergo a metamorphosis in the course of their development and as the patient ages. Early atherosclerotic lesions begin with lipid infiltrates and are highly dynamic lesions containing inflammatory cells (monocytes, leukocytes, and macrophages); inflammatory cytokines (interleukins such as IL-1β and IL-6; tumor necrosis and growth factors such as TNF-α and TGF-β1; and metalloproteinases such as MMP-2 and MMP-9); oxidation products (e.g., oxy-LDL cholesterol); and in diabetic patients, glycation products [34,37]. Atherosclerosis is not solely initiated by atherogenic lipoproteins such as Lp(a), LDL, IDL, or VRDL, but also by genetic factors, such as short non-coding nucleotide sequences of microRNAs [37,38,39,40,41]. These microRNAs play a role in the regulation of all metabolic pathways through the selective blocking of mRNA, which then affects the production of proteins and enzymes [42].

Further development of the atherosclerotic plaque involves both the expansion and breakdown of the matrix, leading to an increase in plaque volume. These processes are regulated by factors that promote the growth of smooth muscle cells and plaque fibrosis (a positive phenomenon that stabilizes it), as well as by factors that digest the plaque, resulting in the formation of necrotic areas within it [43]. Both lipid and necrotic nuclei, along with the digestion of the fibrous matrix with a thin fibrous cap formation, can increase the risk of atherosclerotic plaque rupture and subsequent thrombus formation, leading to sudden arterial occlusion [43].

Plaque rupture and thrombotic occlusion are typical in coronary arteries; however, they are less commonly seen in internal carotid artery stenosis (ICAS) and account for only 10–20% of ischemic strokes [44,45]. In the case of ICAS, a more common etiology of stroke is the embolization of cerebral arteries with detached fragments of atherosclerotic plaque (seen in approximately 70–80% of strokes associated with ICAS) [46]. Risk factors for cerebral ischemia associated with atherosclerotic plaque include inflammation within the plaque (e.g., MMP-9 concentration predicts the occurrence of cerebral ischemia), large plaque surface area (over 40 mm^2^), ulceration of its surface, a high lipid-necrotic load, and the presence of plaque vascularization (angiogenesis) [46,47].

Calcification is the final stage of atherosclerotic plaque development, with TNF-α contributing to this process by activating the mineralization of extracellular matrix and osteogenic differentiation [48]. Uniform calcification of the plaque is an element of metabolic ‘silencing’ (plaque stabilization); however, focal, scattered calcifications are unfavorable phenomena because they increase tension (shear stress) within the atherosclerotic lesion.

Intravascular imaging tools such as optical coherence tomography (OCT) and intravascular ultrasound (IVUS), as well as imaging of the atherosclerotic plaque using CT angiography (CTA) and magnetic resonance angiography (MRA), allow us to detect the coexistence of atherosclerotic lesions at various stages of development in an individual patient [49,50,51,52,53]. Necrotic lipid changes in atherosclerotic plaques, which may be ulcerated or even micro-ruptured, are not uncommon and may heal over time. The presence of unstable plaque features does not necessarily indicate an acute ischemic event. Therefore, it is important to intensify the management of cardiovascular risk factors to reduce the chances of such an event occurring. To prevent the progression of atherosclerosis in its initial stages, it is reasonable to use more intensive interventions, including both pharmacological and surgical methods. However, in cases where the condition has already advanced and the lesions are considered ‘old’ and ‘silent’, guidelines recommend a different approach [54,55,56,57].

### 3.2. Differences in the Morphology of Atherosclerotic Lesions in Elderly People

In the coronary and lower limb arteries, metabolic activity and dynamics of atherosclerotic lesion formation are greatest in young patients but decrease with age. It is important to realize that in individuals under 70 years of age, the pattern of atherosclerotic changes in the coronary arteries differs from those found in older patients [58]. Studies indicate that individuals within the age bracket of 90–99 years have a similar number of atherosclerotic plaques as well as a degree of flow reduction when compared to those within the age bracket of 60–69 years. However, atherosclerotic plaques from individuals in their 90s show more fibrotic, stable changes that are silent or resting, with signs of regression. Additionally, these plaques exhibit fewer lipid and active inflammatory changes when compared to those present in individuals aged 60–69 years [58].

Similarly, changes in the superficial femoral artery caused by atherosclerosis progress at a slower rate. Plaque growth is slower, and there is less development of plaque vascularization. Additionally, the presence of lipid-necrotic elements is reduced, there is a greater fibrotic component, and there is greater proliferation of plaque smooth muscle cells [44]. It is more common to encounter compensatory positive remodeling of the vessel along with collateral circulation. This results in the late occurrence of intermittent claudication and relatively rare occurrence of lower limb critical ischemia [44].

Renal artery stenosis (RAS) is more common in individuals aged 65–70 years and above [59,60,61]. In RAS, plaques are relatively young, and their structure is characterized by high development dynamics. This results in the progression of renal failure, poorly controlled hypertension, episodes of heart failure (HF) exacerbations, and ischemic chest pain not corresponding to the condition of the coronary vessels, as well as increases the risk of mortality, cerebrovascular accidents, and episodes of acute HF [59,60].

These differences in the morphology and dynamics of atherosclerotic lesion development in the coronary circulation and arteries of the lower limbs (peripheral arterial disease—PAD) are associated with a lower risk of acute ischemic syndromes in the elderly when compared to younger patients, even though the incidence of atherosclerosis is similar in both groups of patients, and despite a decline in the ability of vessels (endothelium, smooth muscle cells) to self-heal and repair after numerous mechanical and biochemical damages [44,58].

Atherosclerotic lesions in the carotid arteries behave differently than those of the coronary arteries, with new atherosclerotic plaque elements being added to already mature (heavily calcified) components [61]. This means that the risk of ischemic stroke associated with atherosclerosis of carotid arteries and the aortic arch increases with age, and that multiple comorbidities common in the elderly result in poor rehabilitation after stroke and an increase in the number of patients with permanent disabilities and dementia [61]. Additionally, the lack of well-developed collateral circulation in the circle of Willis is another risk factor for ischemic stroke [62,63,64]. Stenoses of 50–70% in the internal carotid arteries should be carefully monitored, while interventions should be performed in stenoses exceeding 70% since recovery after a stroke in this group of patients is difficult (the risk of stroke involving arteries supplying the brain does not disappear with age).

Stenosis in the renal artery is a strong indicator of increased mortality and is a risk factor for CVD, MI, stroke (major cardiac and cerebral events—MACCE), and HF. However, the potential benefits of revascularization are unclear, as results have been suboptimal [65].

To summarize, it is crucial to minimize inflammatory and pro-atherosclerotic processes. A greater emphasis should be placed on ‘hardening of the vessels’ affected by atherosclerosis and improving arterial compliance to improve the prognosis, reduce symptoms, and strengthen the blood supply to target organs (brain, kidney, lower limb muscles, etc.) [66,67,68,69]. Surgical and endovascular interventions should be reserved for emergencies and life-threatening situations. Balanced pharmacotherapy is associated with a reduction in side effects and drug interactions, while careful referral of patients for surgical and endovascular interventions helps to avoid peri-operative complications and those related to post-operative pharmacotherapy (e.g., antiplatelet and antithrombotic treatment) [70,71,72]. There is still uncertainty as to whether some pharmacological interventions should not be guided individually. There could be a potential for employing artificial intelligence and machine learning systems to guide an optimal timing for medical treatment initiation [54,55]. Furthermore, it can be unclear whether certain patient populations, such as older adults and those with multiple chronic medical conditions, will derive benefit from an intervention. Frailty is an assessment tool that can be used to guide decision-making process for older patients [56]. In elderly patients, the following are observed more often: inferior wound healing, bleeding and thromboembolic events, renal complications, a more difficult return to daily activities, deterioration of cognitive functions and dementia, presence of chronic diseases, and frailty syndrome. All of these factors increase the risk associated with interventional procedures as well as complications in the post-operative period [73,74,75,76,77].

## 4. Epidemiology, Risk Factors, and Clinical Course of Atherosclerosis in Arterial Territories outside Coronary Arteries, with a Particular Emphasis in Elderly Patients

According to data from the Central Statistical Office, there were nearly 10 million people in Poland over the age of 60 years in 2021, including approximately 120,000 individuals aged over 90 years. This number is constantly increasing due to advancements in medical technology, better living standards, and increased awareness regarding health in society. With time, the prevalence of atherosclerosis also rises, meaning that the management of elderly patients with this condition is becoming increasingly challenging.

Patients with atherosclerosis in one of the arterial areas following MI, limb ischemia, or cerebrovascular accident have a high risk for the coexistence of atherosclerotic stenoses in other arterial areas [78,79]. Epidemiological studies indicate that among middle-aged patients admitted to angiography with one-territory atherosclerotic disease, about 1.5% have arterial occlusive lesions in all arterial beds (CAD + ICAS + PAD, and/or RAS) [78]. This number increases to 4% in patients aged 80 years old [79]. The relative risk of having at least two-territory atherosclerotic occlusive disease is 15.7-fold higher in patients with claudication; 2.1-fold in patients with multivessel CAD; 2.8-fold for serum creatinine level >1.3 mg/dL; and 1.9-fold, 2.4-fold, and 2-fold in patients with hyperlipidemia, smokers, and women, respectively [78]. One ischemic event leads to another in the same or another arterial area [46,80,81]. In the EXSCEL trial including 14,751 participants; 26.5% were without atherosclerosis, and 58.9% had one-bed, 12.3% had two-bed, and 2.3% had three-bed disease [82]. An increasing burden of atherosclerotic disease was associated with increasing risk of major adverse cardiac events (HR, 1.71 [95% CI 1.46–2.02]; 2.61 [2.17–3.15]; and 3.46 [2.69–4.45] for one, two, and three beds, respectively, *p* < 0.001 for all) and all-cause mortality (1.94 [1.56–2.42]; 3.03 [2.33–3.95]; and 3.66 [2.59–5.18] for one, two, and three beds, respectively, *p* < 0.001 for all) [82]. In line, in the REACH registry including 23,985 participants, one-year outcomes of the composite cardiovascular death, nonfatal myocardial infarction, and nonfatal stroke event rate was 4.3% for the overall population and highest in patients with triple-bed disease (9.9%) [83].

Its worth noting that clinicians typically treat only the initially affected territory, mainly CAD, without consideration of the other affected territories, and they may lack awareness of the overall atherothrombotic syndrome [84].

Atherosclerosis in coronary and non-coronary arteries is stimulated by the same cardiovascular risk factors (i.e., age, male, cigarette usage, diabetes, hypertension, dyslipidemia, and obesity) [46,85,86]. However, in the case of ICAS, the dominant factor is hypertension, while in coronary arteries, the dominant factor is hyperlipidemia. In the Framingham Heart Study, for every 20 mmHg increase in systolic BP, there was a doubling in risk of developing ICAS greater than 25% [49,57]. Smoking and diabetes are the main factors in PAD (Table 1) [87]. A meta-analysis of epidemiological studies from the USA, including a total of over 38,000 participants covered by the primary prevention program for heart and vascular diseases, showed that cigarette smoking and diabetes increase the risk of developing PAD by 4.0–5.5 times and that this risk is 10 times greater in females when compared to males [86]. Diabetes is associated with additional risks for PAD patients such as diabetic foot, diabetic angiopathy, sensory neuropathy, and hyalinization of the intima of small arteries below the knee [88,89]. Furthermore, in diabetes, microangiopathy coexists with macroangiopathy, amplifying cardiovascular risk in the patient [86]. Thus, for patients with diabetes, to obtain more systematic clinical care, comprehensive diabetes care centers focusing on panvascular diseases are required [90].

### 4.1. Familial Hypercholesterolemia and the Extent of Atherosclerotic Lesions

Familial hypercholesterolemia (FH) is a monogenic, autosomal dominant disorder that from birth results in elevated low-density lipoprotein cholesterol (LDL-C) and markedly increased risk of premature atherosclerosis [91,92]. In this disorder, the metabolism of LDL-C is altered through mutations in the gene for LDL receptor (LDLR) and less commonly in those for apolipoprotein B (APOB), proprotein convertase subtilisin-kexin type 9 (PCSK9), and others [93]. In most FH cases, the mean LDL-C level exceeds 190 mg/dL in adults and over 160 mg/dL in children [94]. According of the Dutch Lipids Clinics Network (DLNC) criteria, FH is diagnosed in 1 out of 250 adults [95]. The increased LDL-C levels since childhood are associated with a premature manifestation of atherosclerotic disease. The risk of developing CAD in FH patients is approximately 13-fold higher than in the general population [96]. CAD is evident in patients with FH from the age of 17 in men and the age of 25 in women, and up to 25% of the adolescents with FH present coronary artery calcification and/or aortic valve calcifications [97]. In a study that included a representative sample of adult patients with hypercholesterolemia in outpatient clinics in Poland, FH based on DLNC criteria was diagnosed in 3.6% of the examined patients [97].

Despite the common belief that atherosclerosis extent is greater and more severe when hyperlipidemia begins at very early stage of life, patients with FH atherosclerotic involvements differ tremendously in terms of arterial territories. Atherosclerotic plaques causing significant obstructive arterial disease mainly affect coronary and lower limb arteries [98,99]. Conversely, in carotid arteries, FH is associated with the increased carotid intima–media complex thickness in common and internal carotid arteries, but it is less likely to cause a significant arterial lumen narrowing [100]. In this line, atherosclerotic plaques are more prevalent in renal arteries in FH, but rarely obstructive, as compared to patients without FH [101]. However, the small number of available studies, as well as their characteristics (sample size, diagnostic criteria used, retrospective or cross-sectional design) limits the evidence for association between FH and stroke, PAD, or RAS [102]. In conclusion, FH is associated with a substantial excess mortality from CAD in young adults but may not be associated with a substantial excess mortality in older patients [102,103].

### 4.2. Lower Extremity Peripheral Arterial Disease

PAD is the third leading cause of atherosclerotic morbidity, following CAD and stroke. PAD affects >230 million adults worldwide and is associated with increased risk of various adverse clinical outcomes [104,105]. Every year in Poland, there are approximately 40,000 hospitalizations due to PAD and over 9000 amputations related to this disease [106]. Various diagnostic methods for detecting PAD have led to conflicting results in epidemiological studies [107,108,109,110]. Some studies suggest that males are slightly more prone to develop PAD than females. A meta-analysis conducted by Lin et al. revealed that the prevalence of PAD is 6% in males and 5% in females aged between 60 and 69 years; the prevalence is 11% and 9% in those aged between 70 and 79 years; and 26% and 21% are over 80 years of age, respectively [107]. The Rotterdam study, which was published in 1998 and based on ankle-brachial index (ABI) measurement, revealed that the incidence of PAD was greater in females than in males, with 20.5% of females and 16.9% of males affected [107]. There is a clear increase in incidence with age, ranging from 6.6% in males and 9.5% in females aged 55–59 years to 52.0% in males and 59.6% in females over the age of 85 years [107,108,109,110].

**Figure 1:**
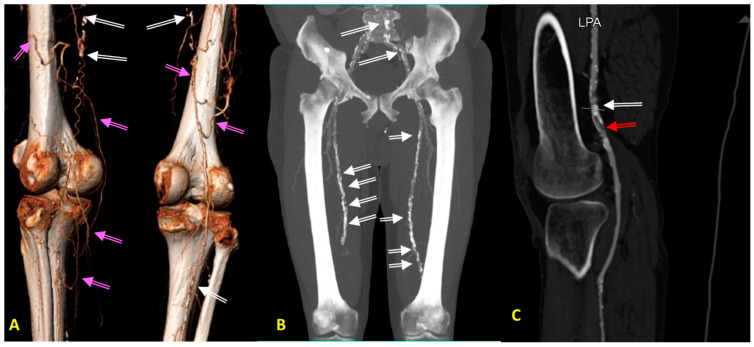
(**A**–**C**) Diagnostic images of peripheral arterial disease (PAD) in patients with advanced atherosclerosis, obtained with computed tomography angiography (CTA). (**A**) CTA image obtained in a 73-year-old hypertensive man with asymptomatic PAD showing long occlusions of both superficial femoral arteries (SFA). Please note a very well developed collateral circulation (pink arrows) from deep femoral arteries and white spots along both SFAs corresponding to calcifications (white arrow). (**B**) CTA image obtained in a 78-year-old hypertensive man with moderately symptomatic PAD showing occlusions of both SFA in the Hunter channel. Please note a very excessive calcifications in iliac and femoral arteries (white arrows). (**C**) CTA image obtained in a 78-year-old hypertensive woman with type 2 diabetes, as well as a critical PAD causing rest pain resulting from ruptured calcified plaque (white arrow) in the left popliteal artery (LPA), with a recent vessel thrombosis below the calcified plaque (red arrow). Please note the lack of collateral circulation.

Such a high frequency of PAD in the elderly is not necessarily correlated with the frequency of intermittent claudication, which affects only 6.0% of males and 2.5% of females at the age of 85 years [109]. This is due to adaptive changes occurring in the limb arteries, such as the slower dynamics of plaque growth, positive vascular remodeling, and the development of collateral circulation (Figure 1). Furthermore, older people with PAD tend to present with atypical symptoms such as general fatigue, leg weakness, tenderness to touch, hypoesthesia, and edema, rather than intermittent claudication. It is also worth noting that symptoms of neuropathy, sciatica and other root pain syndromes, venous thrombosis, and muscle atrophy during lipid-lowering therapy can often mimic PAD symptoms and should, therefore, be considered in the differential diagnosis.

In an outpatient setting, the probability of PAD in individuals who are under the age of 80 years can be estimated using the online calculator (http://ckdpcrisk.org/padrisk, accessed on 9 January 2024). It is difficult to predict the risk of incidents and death in PAD patients due to its poor symptomatology. However, after PAD diagnosis, an estimated 20% of patients may experience MI, stroke, or death within the first 5 years. Critical ischemia of the lower limb resulting in amputation has the highest risk of death, and after amputation, the expected average survival time for 60% of patients is 2 to 5 years [106,107,108,109,110].

Smoking is the greatest modifiable risk factor for the development and progression of PAD. According to Meijer et al.’s study, smoking accounted for most occurrence of PAD cases with odds ratio of 2.8 (95% CI, 2.3–3.4, *p* < 0.001) [84]. Tobacco smoke increases endothelial cell permeability by the formation of reactive oxygen species, which enables atherosclerosis to settle within vessel walls [111].

It is important to note that patients aged 65 years with PAD (ABI < 0.9) have a 2.43-fold greater risk of death (95% CI: 2.18–2.72) compared to those without PAD (ABI ≥ 0.90). However, as PAD patients age beyond 65 years, mortality rates decrease with an average reduction of 13% (95% CI: 0.83–0.92) for each additional 5 years [107]. The authors of the study concluded that with increasing age, PAD gradually ceases to be a risk factor for death when compared to the same age group without PAD [107].

The results of a Japanese cohort study, which followed 3122 PAD patients over 65 years of age, showed that the number of cardiovascular risk factors is an important parameter determining the survival of patients [110]. In this study, the accumulation of three cardiovascular risk factors (diabetes, hypertension, hyperlipidemia) occurring in 419 (13.4%) patients increased the risk over threefold for both MACCE (HR: 3.06; 95% CI: 2.45–3.82; *p* < 0.0001) and critical lower limb ischemia (major adverse limb event (MALE): HR: 3.22; 95% CI 2.39–4.35; *p* < 0.0001). The age group itself (65–74 years, 75–84 years, >85 years) was not a risk factor for MACCE; however, greater age was a protective factor against the occurrence of MALE, which is associated with cessation of inflammatory-thrombotic processes in the lower limbs. Both middle-old seniors (75–84 years old) and oldest-old seniors (>85 years old) had a lower risk of MALE (HR 0.79; 95% CI 0.66–0.96, respectively; *p* = 0.0150 and HR: 0.53; 95% CI 0.36–0.77; *p* = 0.0010) when compared to the group of youngest-old seniors (65–74 years old) [110].

Sykora et al. assessed the risk of MACCE in more than 22,000 outpatients with PAD according to age structure (<50, 50–59, 60–69, ≥70 years) and ABI (<1.0; 1.0–1.4; >1.4) [112]. MI and stroke were significantly more common in PAD patients diagnosed before the age of 60 years (10%) compared with patients whose PAD was diagnosed after the age of 70 years (6.8%) (HR 2.33; 95% CI 1.95–2.78) [112]. Similarly, the risk of MALE and amputation was lowest in the group of patients over 70 years of age and gradually increased in the groups of younger patients. It is puzzling that the causes of death were not analyzed in this study, whereas the mortality rate after 2 years of observation was 51% [112].

Another study investigated the factors that influence the risk of death in PAD patients over the age of 80 and 90 years [113]. One-year mortality was 25% (35/123 patients) above the age of 80 years compared to 10% (13/123 patients) in the group of patients under the age of 80 years. In the group of PAD patients aged 80 years and above, it was found that the concentration of cardiovascular markers such as troponin > 40 ng/L (HR: 4.6; 95% CI: 1.4–15.3) and NT-pro-BNP > 450 pg/mL (HR: 3.9; 95% CI 1.8–8.8) and the occurrence of MALE (HR: 3.1; 95% CI 1.6–5.9) were strong predictors of patient death [113]. Features of ischemia, HF, and MALE determine the survival of this group of patients.

The coexistence of atherosclerosis and diabetes is associated with the worst short- and long-term prognosis and poses significant problems in the diagnosis and management of these patients. Diagnosis of PAD by measurement of ABI (class I-C of the recommendations for PAD diagnosis) is characterized by a sensitivity and specificity of >90–95% when ABI < 0.9 [114]. Ankle-brachial index < 0.5 is an indicator of severe limb ischemia. However, this indicator often fails in patients with diabetes or end-stage renal failure and in those being treated with dialysis [114]. Hyalinization of the vessel intima leads to a falsely high ABI (above 1.4–1.5), making PAD diagnosis difficult. Atherosclerosis of small vessels in the tibial, peroneal, or dorsal foot and metatarsal arteries makes the treatment of these patients very difficult and is a challenge for vascular surgeons. In these patients, the toe-brachial index (TBI) is assessed using a small cuff placed on the big toe and an ultrasound (class of recommendation: I-C). A TBI value < 0.7 is diagnostic for PAD [106]. In elderly patients, due to the frequent coexistence of diabetes and renal failure, the TBI index should be used more often to determine the prognosis.

According to most scientific societies, in-depth diagnostic imaging (duplex Doppler ultrasound, angio-CT, angio-MRI, angiography) of the type, location, and severity of stenoses in the lower limb arteries should only be performed in elderly patients with features of critical or acute lower limb ischemia. This diagnosis should be justified by the need for revascularization of the lower limb artery or arteries in patients with ulceration or trophic changes and in those who are at risk for amputation [115]. Imaging tests with contrast administration are particularly problematic in the elderly, who are characterized by multimorbidity and are associated with the risk of renal function deterioration, up to and including dialysis [115].

Patients presenting with symptoms of MALE, as well as with ischemic rest pain (Fontaine class III symptoms), ulceration or gangrene (Fontaine class IV), or Rutherford class 4–6 symptoms, require revascularization to save the limb. This is particularly relevant in non-healing ulcers despite previous treatment. If the systolic BP in the toe is less than 80 mmHg, the likelihood of healing the ulceration is low [106,107,116]. Treatment may be considered on an individual basis in patients with short-distance isolated arterial stenosis with significant distance limitation (claudication in rest or after short-distance < 50 m, no improvement after hardening with walking—according to Rutherford class 3) [107].

Acute limb ischemia unrelated to atherosclerosis should always be kept under consideration [116]. Ischemia of the lower limb may be caused by embolism of the femoral artery, popliteal artery, or tibiofemoral trunk (as in the course of atrial fibrillation), or by vegetation from the heart valve or left atrial myxoma. Such patients require urgent embolectomy regardless of their age.

### 4.3. Renal Artery Atherosclerosis

Renovascular disease (RVD) due to atherosclerotic uni- or bilateral RAS is a disease of the elderly and generally involves the proximal segment of the renal artery. Thus, in the elderly, RVD is a de novo phenomenon, and the plaque is considered as ‘young’. The significance and treatment of RAS are some of the most controversial issues. RAS is predominately an age-dependent pathology, having a prevalence of less than 1% in the general population. However, above the age of 65 years, its incidence is 7%, while above the age of 75 years, it can be found in approximately 40% of examined patients [117].

If RAS significantly reduces renal perfusion and causes renal ischemia, it leads to activation of the renin–angiotensin–aldosterone (RAA) axis with the following consequences: increased peripheral resistance, water and sodium retention, hypervolemia resulting in the development of hypertension, myocardial hypertrophy, and accelerated atherosclerosis. The consequences of RAS include MI, stroke, aortic dissection, and pulmonary edema [118,119,120]. A cardiac manifestation of RAS may be circulatory failure. In its acute form, RVD manifests as pulmonary edema with preserved left ventricular systolic function (pulmonary flash edema), while in the chronic form, this is seen as left ventricular HF. Chronic renal ischemia results in nephropathy and progressive failure, combined with renal atrophy up to and including cirrhosis [118,119,120]. In unilateral RAS, prolonged stimulation of the RAA axis; increased sympathomimetic activity; oxidative stress; and activation of endothelin, thromboxane, and 20-hydroxyeicosatetraenoic acid (20-HETE), with a concomitant decrease in prostaglandin and nitric oxide production, can lead to inflammation, fibrosis, and progressive failure of the second kidney [121]. Historical studies show that the four-year survival rate of RAS patients with over 75% stenosis is 68%, while those with 95% stenosis is 48%, compared to 89% in patients without this pathology [122].

RAS is the most common cause of secondary hypertension, so it is not surprising that it can be detected in approximately 2–5% of hypertensive patients and in 30–40% of patients with coexisting hypertension and renal failure. Moreover, RAS is also found in 30–50% of patients with congestive HF. Finally, RAS increases the risk of ischemic heart disease and PAD by 4 times, HF by 3.5 times, stroke by 3 times, and death by 2.6 times [123]. In patients with coronary artery disease (CAD), the incidence of RAS is approximately 30–33%, of which 7–15% of these patients have a stenosis of greater than 50%. Bilateral stenosis is found in 4–11% of patients [118,124]. The prevalence of RAS is estimated to be as high as 40% in patients with PAD and as high as 50% in patients with multilevel atherosclerosis [125,126]. It is noteworthy that in the group of patients with incidentally detected RAS at the moment of diagnosis, 65.5% had hypertension and 27.5% had renal failure [127].

Atherosclerotic RAS can be suspected in patients with a sudden onset of hypertension over 50–55 years of age [128]. Additionally, this should be suspected in essential hypertension patients who have a worsening of previously well-controlled BP [128]. Other features that may indicate the presence of RAS are unexplained renal failure or its onset after the initiation of angiotensin-converting enzyme inhibitors (ACEIs), angiotensin receptor blockers (ARBs), or sodium-glucose cotransporter-2 inhibitors (SGLT2i); a difference in the size of kidneys by more than 1.5 cm; resistant or malignant hypertension; recurrent pulmonary edema with preserved left ventricular contractility; or symptoms of multilevel atherosclerosis [128]. Age, female sex, hypertension, presence of generalized atherosclerosis, multivessel CAD, diabetes, and elevated creatinine and LDL cholesterol levels have all been identified as independent risk factors for RAS in multivariate analysis [129,130].

**Figure 2:**
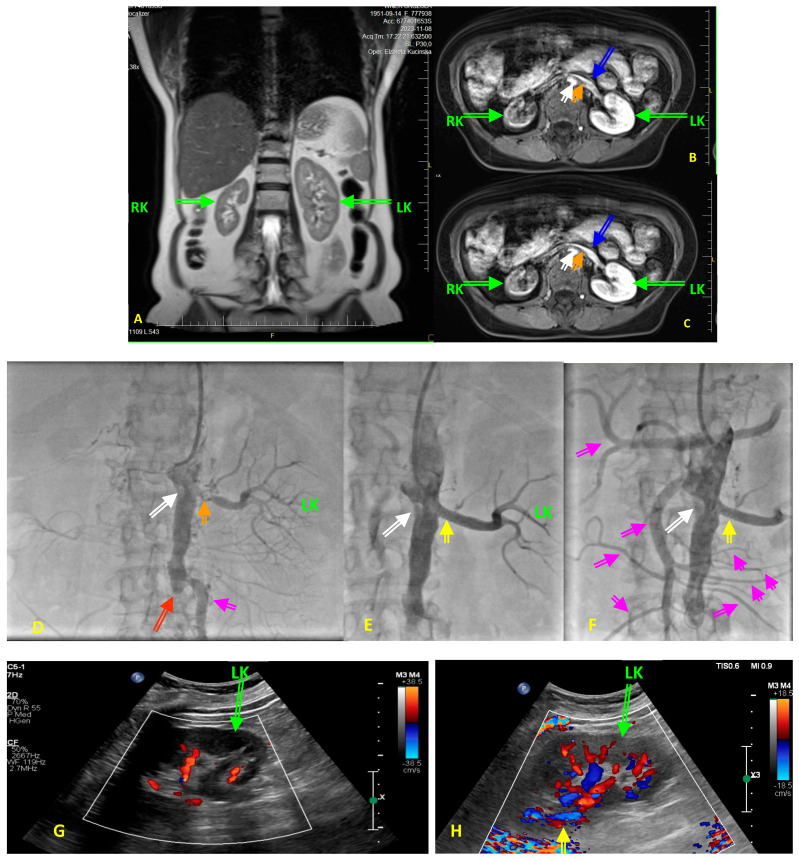
(**A**–**H**) Diagnostic images obtained in a 73-year-old hypertensive woman with type 2 diabetes before renal artery angioplasty with stent implantation (PTA) for the left renal artery stenosis (RAS). The patient was referred to renal PTA because of resistant hypertension, with blood pressure values above 200/120 mmHg despite five blood-lowering agents in maximally tolerated doses, after the patient developed acute renal failure following treatment initiation with empagliflozin. At the six-month follow-up period following PTA, the patients was still on five blood lowering agents with blood pressure values not exceeding 150/75 mmHg, with a stable renal function with an eGFR of 27 mL/min/1.73 m^2^. (**A**) Pre-intervention diagnostic magnetic resonance angiography (MRA) showing quantity alterations in right kidney (RK) and left kidney (LK) dimensions and unequal renal parenchymal signal intensity. (**B**,**C**) Transverse scans of the aorta, renal arteries, renal veins (blue arrows), and both kidneys on MRA. Images display, in early and late gadolinium-contrast phases, the small size of the RK, low signal in the RK, and unclear corticomedullary boundary in both kidneys. Please note the intensive calcifications in the abdominal aorta (white arrow), and the critical non-calcified, presumably soft ostial lesion of the left renal artery (orange arrow). Perfusion of the LK is preserved as yet, while the RK shows features of cirrhosis. (**D**) Renal catheter angiography confirmed an ostial 95% left RAS (orange arrow), and absent right renal artery (white arrow). Thus, the patient is presented with a stenosis of a single functional kidney. Please note the total occlusion (red arrow) of the distal part of abdominal aorta and both common iliac arteries. Pink arrow indicates collateral pathway. (**E**) The final result of PTA in renal artery (yellow arrow). Please note that the PTA procedure was performed via a transradial access route due to the total occlusion of the distal part of abdominal aorta and both common iliac arteries (Leriche’s syndrome). Pink/violet arrow indicates the large collateral artery providing blood supply to the pelvis and lower extremities. White arrow is for the total occlusion of the right renal artery. (**F**) Final angiography of aorta displaying numerous collaterals (pink arrows) from cephalic trunk, mesenteric arteries, to abdominal and pelvis organs. Yellow arrow indicates the final effect of RAS procedure, whereas white arrow desplays occluded right renal artery. (**G**) Color Doppler ultrasound showing reduced intrarenal flow in the LK before PTA. (**H**) Color Doppler ultrasound showing restoration of the intrarenal flow in the LK after PTA, yellow arrow indicates the final effect of RAS procedure.

Diagnosis for RAS can be started with ultrasonography of the renal arteries (Class IB recommendation). Compared to the carotid or lower limb arteries ultrasonography, this examination is more challenging, as it requires excellent equipment and an experienced examiner. However, it allows for accurate RAS detection exceeding 60% with a sensitivity and specificity of 98%, as well as with a positive predictive value of 99% and a negative predictive value of 97% [131]. Among the most important ultrasonographic parameters that could indicate the presence of RAS are peak systolic velocity (PSV) and diastolic velocity at the stenosis site exceeding 2.0 and 0.5 m/s, respectively, and the renal-aortic ratio (RAR) greater than or equal to 3.5 at the stenosis site [131,132]. The examination is complemented by parameters of renal size, structure, and assessment of intrarenal flow. Preoperative assessment of the degree of RAS is essential for determining the treatment options (Figure 2). CTA is characterized with high sensitivity; however, CTA carries a risk of radiation exposure, requires exogenous contrast agents that are potentially nephrotoxic, and cannot be performed in patients allergic to iodine contrast agents [133,134]. In contrast, MRA has the benefits of no ionizing radiation, high repeatability, and a low incidence of adverse reactions with gadolinium contrast agents [134]. MRA for the preoperative examination of the kidney that has been increasingly studied as an MRI technique has continued to advance [135]. Both CTA and MRA have known limitations as they tend to overestimate the degree of stenosis and, in addition, large calcifications make it difficult to assess the degree of stenosis. On the other hand, renal perfusion scintigraphy and renin activity in plasma or renal veins, which were commonly used years ago, are not currently recommended as diagnostic methods (Class III C recommendations) [136].

Non-surgical management of patients with RAS is identical to that for atherosclerosis localized to other arterial areas and includes a rigorous control of risk factors through smoking cessation, diet, weight normalization, exercise, and pharmacotherapy to normalize BP, achieve target blood lipid levels, and manage diabetes [137,138,139,140,141,142,143,144,145,146,147].

In the case of RAS, the largest challenge is to determine whether the existing stenosis is an incidental finding or whether it significantly impairs renal perfusion and is responsible for the existing problems, therefore being an indication for intervention. This can be particularly difficult in elderly patients, as they generally have advanced atherosclerosis in various arterial areas; impaired renal function with reduced eGFR; and very often, long-term hypertension.

Currently, the treatment of choice for RAS is percutaneous transluminal angioplasty (PTA), generally combined with stent implantation, which significantly reduces the incidence of recurrent stenosis and improves long-term outcomes [120,141,146,148,149]. It has a low complication rate of less than 5% in experienced centers and an acceptable stenosis recurrence rate of approximately 15% [148,149]. Invasive treatment aims to improve BP control and protect renal function, which should translate into an improved prognosis for the patient. The results of numerous observational studies (usually single-center) indicate an improvement in BP control in approximately 60–70% of patients with atherosclerotic RAS, although curative rates are only 5–15% of patients. Improvement in renal function is seen in approximately 25–30% of patients and is as common as a deterioration in renal function. In approximately 40–50% of patients, no significant effect of the procedure on renal function is observed, which can be interpreted as both a lack of effect and a favorable stabilization of renal function [149,150]. Randomized clinical trials (RCTs) have compared the effects of PTA and pharmacological treatment on BP control, renal function, cardiovascular incident rates, and patient survival. Of the eight completed RCTs, only the two small ASPIRE 2 and RENAISSANCE trials showed the superiority of PTA over pharmacological treatment, and this was only regarding BP, with no effect on renal function, MACCE rates, or survival [150]. Other studies, including the two largest, the ASTRAL and CORAL studies, did not show an advantage of invasive procedure over pharmacotherapy in any of the parameters analyzed [142]. Although a meta-analysis of four of these trials showed a significant improvement in diastolic BP and a reduction in the number and doses of blood-lowering drugs after PTA compared to pharmacotherapy, the lack of effect of PTA on prognosis in the RCTs remains a fact [150].

As a result, the current guidelines allow PTA for atherosclerotic RAS only in cases of unexplained recurrent HF or sudden pulmonary edema and having the lowest possible recommendation level of IIb-C [136]. Indeed, the mortality rate of patients with flash pulmonary edema at the six-year follow-up was 90%, and in cases of recurrent chronic circulatory failure, performing PTA reduces the number and frequency of hospitalizations and improves the NYHA class, which fully justifies this treatment method [151,152].

The guidelines have a gap in terms of several important clinical situations. In the case of true resistant hypertension (4–5 hypotensive drugs at maximum doses including two diuretics) coexisting with RAS, performing a PTA procedure to improve pressure control seems fully justified. Bilateral stenosis or stenosis of a single functional kidney was omitted from the guidelines. In our experience, performing PTA in these patients improves BP control in 80% and renal function in 40% of patients [153,154]. Finally, patients with RAS and rapidly progressive nephropathy manifested by a systematic rapid decline in eGFR remain a challenge. The five-year survival rate among these patients is 25% in those receiving pharmacotherapy alone. However, in those treated with combined PTA and pharmacotherapy, the five-year survival rate increases to 90%, which we believe is a strong indication for intervention [152]. Our study demonstrated that if an increase in eGFR of 11 mL/min/1.73 m^2^ is achieved as a result of the procedure, which occurs in one-third of patients, this results in a significant reduction in death by 58% and MACCE by 46% at the four-year follow-up [154]. In contrast, a reduction in systolic BP of more than 20 mmHg (which was achieved in 46% of patients) or diastolic BP ≥ 5 mmHg (24% of patients) results in a 90% reduction in stroke incidence [154].

Factors that may indicate a potentially beneficial effect of the procedure include the short (less than 2 years) duration of hypertension, very elevated levels of BP, resistant hypertension, deterioration of renal function recently or after the initiation of ACEIs/ARBs, episodes of acute or chronic circulatory failure with preserved left ventricular contractility, high degree of stenosis, and bilateral stenosis or unilateral stenosis of a single functional kidney. Patients with suspected or established RAS should be referred to a specialist center with appropriate experience in the assessment of these patients and invasive treatment.

### 4.4. Carotid Artery Atherosclerosis

The prevalence of asymptomatic ICAS also increases with age. Stenosis above 50% was found in 4.8% (95% CI: 3.1–7.3%) of males and 2.2% (95% CI: 0.9–4.9%) of females under 70 years of age, compared with 12.5% (95% CI: 7.4–20.3%) in males and 6.9% (95% CI: 4.0–11.5%) in females over 70 years of age [155]. Under 50 years of age, the incidence of ICAS > 50% is minimal (0.2% in females and 0.3% in males).

Histopathological findings from carotid artery atherosclerotic plaques clearly indicate that as the patient’s age increases, atherosclerotic lesions become larger, containing locally more inflammatory cells and MMP-9 and fewer smooth muscle cells (which normally stabilize atherosclerotic plaques) [156]. These characteristics of carotid atherosclerotic lesion structure predispose the patient to symptoms of cerebral ischemia, the risk of which increases with the patient’s age. The result is that the risk of death and stroke in patients over 75 years of age is greater if the patient is only on pharmacotherapy compared to those with additional surgical treatment [157]. This is true despite the current availability of effective pharmacotherapy [156,157,158,159,160,161].

The recommendations include pharmacotherapy, smoking cessation, and dietary modification in all patients with asymptomatic as well as symptomatic ICAS, as well as revascularization in selected groups of patients (endarterectomy (CEA) or PTA). Pharmacotherapy, as in other arterial areas, includes hypotensive, antiplatelet, hypoglycemic, and lipid-lowering agents. A prospective cohort of 101 patients with asymptomatic ICAS found lower annual risk of ipsilateral stroke events (AR = 0.34%, 95% CI 0.01–1.87) when patients were treated with antiplatelet agents, statins, and anti-hypertensive medications [162]. A systematic review of 3724 patients with asymptomatic ICAS showed decreasing rates of stroke related to improvements in medical therapy [163].

Despite medical treatment, asymptomatic ICAS exceeding 70% is associated with an annual risk for ischemic stroke of 2–4% (10–20% over 5 years) in the elderly [157]. In contrast, the presence of neurological symptoms in patients without interventional treatment (CEA or PTA) increases the risk for developing stroke by 4–12% per year.

**Figure 3:**
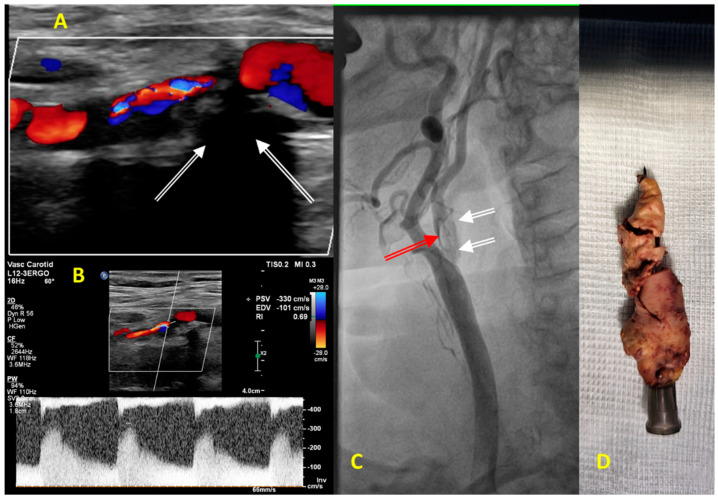
(**A**–**C**) Diagnostic images obtained in an 83-year-old man before carotid endarterectomy on the right internal carotid artery (RICA) stenosis. (**A**) Color Doppler ultrasound showing abnormal turbulent flow in the proximal segment of the RICA. Carotid plaque is hyperechogenic, massively calcified with a typical shadowing below calcifications (white arrows). (**B**) Pulse Doppler showing a significant increase in the peak systolic and the end-diastolic velocities of 330 cm/s and 101 cm/s, respectively, consistent with severe stenosis of the proximal segment of RICA that corresponded to lumen stenosis of more than 90%. (**C**) Carotid catheter angiography confirmed a 2 cm long 95% RICA stenosis (red arrow) with calcified morphology (white arrows). (**D**) The image displays massively calcified plaque excised from the RICA during carotid endarterectomy.

In contrast to other arterial areas, in ICAS, age does not reduce the risk of stroke and death but carries with it additional complications such as severe calcification (Figure 3), vessel tortuosity, stiffness, low compliance, atherosclerosis, and deformation of the aortic arch, which should be considered when treating these patients surgically [164,165].

When deciding on surgical treatment in elderly patients, contraindications and factors increasing the risk of surgical complications should be taken into account [164,165]. Factors that increase the risk of complications from CEA include anatomical features of the stenosis (history of radiation and surgical treatment of neck tissue or previous CEA, tracheostomy, contralateral laryngeal nerve palsy, contralateral carotid artery occlusion, thrombus, stenosis of the distal ICAS or proximal common carotid artery) and the patient’s general condition (age > 80 years, MI in the last 30 days, NYHA class III and IV, severe renal failure or lung disease, recent percutaneous coronary intervention requiring antiplatelet therapy). Risk features associated with stent implantation include unfavorable anatomical conditions for stent insertion such as arterial tortuosity, lack of access site, inconvenient aortic arch, atherosclerosis/aneurysm/malformation of intracerebral arteries, severe carotid artery calcification, thrombus, age over 80 years, severe renal failure, stroke within the last 4–6 weeks, and contraindications to antiplatelet therapy [164,165].

When considering periprocedural complications of PTA and CEA in patients younger than 70 years, the risk of stroke, death, or MI was similar for both methods, while among patients over 70 years, the 30 day risk of stroke and death was elevated for PTA (OR 2.20, 95% CI 1.47–3.29) [166,167]. Based on this, current guidelines state that it is reasonable to consider patient age in choosing between CEA and PTA, as CEA is associated with improved outcomes compared to PTA for those over 70 years, while in younger patients, PTA is equivalent to CEA in terms of risk periprocedural complications, a Class IIa recommendation [167]. In patients undergoing PTA or CEA for either symptomatic or asymptomatic ICAS, perioperative mortality, stroke rates, and other perioperative complications increase in those above 75 years of age, especially above 85 years of age, compared to younger patients [167].

There are limited data on outcomes for frail individuals who undergo CEA or PTA. In a cohort study that included 37,875 patients, with an average age of 75, who underwent either CEA or PTA in the NSQIP database, 27% were frail [168]. In total, 95.4% underwent CEA and 4.6% underwent PTA. Compared to those who were not frail, individuals identified as frail were more likely to experience poor outcomes such as a post-operative complication (OR 3.2 (2.1–4.8) *p* = 0.01) and mortality (OR 2.1 (1.6–3.7) *p* = 0.01), irrespective of the method used [168].

At the same time, good surgical outcomes (CEA and CAS) have been documented in groups of older patients who were carefully prepared for surgery, with particular attention to renal function, anemia, and surgical technique [169,170,171]. In a study conducted by four U.S. centers, the 30 day risk of death and stroke after percutaneous ICAS treatment was 2.8% (11/389) in a group of patients aged over 80 years (83.2 ± 2.8 years), of whom 2/3 were asymptomatic and 1/3 presented with symptoms of cerebral ischemia before the procedure [171]. In a study involving 193 patients ≥75 years of age with symptomatic ICAS, the 30 day risk of death or stroke was 7%, while in asymptomatic ICAS, this risk was 1.7%, and finally, this risk was 1.9% in younger individuals [172].

An extremely important clinical aspect in patients with ICAS, even in those with an asymptomatic course (without features of TIA or stroke: limb paresis, aphasia, facial nerve palsy, amaurosis fugax, etc.), is the progressive deterioration of cognitive function up to and including dementia [173]. It is estimated that there are currently approximately 50 million people living with dementia worldwide and that this number will triple by 2050 [174]. One of the most important factors contributing to the development of dementia is hypertension, particularly isolated systolic hypertension [174,175,176]. Hypertension also causes a very rapid progression of atherosclerosis in the carotid and cerebral arteries. These mechanisms, such as reduced blood supply to the brain due to hemodynamically significant ICAS, systematic but clinically silent embolization of the cerebral arteries, and damage to the blood–brain barrier by excessively elevated systolic pressure (>160 mmHg) result in systematic cognitive decline over months and years [177]. This cognitive decline is initially mild and can be in the form of vascular dementia and/or having a mixed etiology such as vascular and Alzheimer’s disease [178].

ICAS, along with hypertension, diabetes, other cardiovascular risk factors, or intracerebral atherosclerosis, contributes significantly to the onset and systematic progression of cognitive impairment [176,177,178]. There is increasing evidence that dementia may be considered the equivalent of cerebral ischemia. Therefore, in patients with ICAS, it is important to prevent systolic hypertension, which is the primary risk factor for ICAS progression as well as dementia [179,180]. Optimal pharmacological intervention should include hypotensive agents and statins for which brain-protective effects against dementia have been demonstrated, such as those which prevent damage to the blood–brain barrier [179,180]. Such agents include the second-generation calcium channel blocker nitrendipine and some ARBs. In a randomized trial by Hu et al. involving 1244 hypertensive patients aged 60 years or older with normal baseline cognitive test scores, the combined use of rosuvastatin (10 mg/day) and telmisartan (40/80 mg/day) had a synergistic effect and was associated with a reduction in the risk of developing dementia by more than 50% over a seven-year follow-up period [180].

Finally, the occurrence of a TIA or stroke in patients with ICAS can greatly exacerbate the development of dementia. There are known cases of rapid recovery of motor function in patients after stroke but also rapid (3–6 months) development of dementia [181]. Therefore, the authors of this review believe that significant ICAS should not be left to its natural course and that every effort should be made to systematically monitor lesions that are not yet amenable to intervention. Carotid artery ultrasound is recommended every 6 months in lesions causing 50–70% stenosis together with lifestyle optimization, diet, and pharmacotherapy, with particular attention to systolic BP control. In addition, GPs, geriatricians, and psychiatrists should use tests such as the mini-mental state examination (MMSE) to assess cognitive function more frequently and to respond to the first signs of cognitive impairment.

Computed tomography (CT) should also be performed for all patients with dementia, as imaging can change management in up to 15% of cases [182]. According to most scientific societies, in-depth imaging diagnosis (duplex Doppler ultrasound, angio-CT, angio-MRI, angiography) of the type, location, and severity of stenoses should only be performed in elderly patients with features of either symptomatic lesions, as well as in critical or acute organ ischemia. The imaging should be justified by the need for intervention of the stenosed artery, or as a guide to modification in pharmacological treatment [183]. Imaging tests with contrast administration are particularly aggravating in the elderly, who are characterized by multimorbidity and are associated with a risk of deterioration of renal function, up to and including dialysis [184]. Furthermore, heavy calcifications in arteries cause errors in the interpretation of images and thus they require modern imaging tools to omit this drawback, like dual energy computed tomography [185] or functional MRI for both assessment of RAS degree and renal perfusion [186].

In summary, the morphological structure of atherosclerotic plaques varies in different vascular areas. The severity of risk factors contributing to the development of atherosclerotic lesions also varies. These various manifestations of atherosclerosis require accurate consideration of its treatment. However, the advanced age of a patient must not disqualify the patient from surgical or endovascular intervention, especially as life expectancy is increasing and will probably exceed 90 years in 2050 [187].

## 5. Ischemic Preconditioning

Ischemic preconditioning is a well-established method of adapting organs to chronic ischemia. It strengthens the peripheral circulation, prolongs the time to symptom onset, and reduces the risk of critical ischemia.

### 5.1. Peripheral Arterial Disease

In patients with intermittent claudication, to determine the distance that can be walked without pain, a standardized treadmill walk test is performed using a treadmill gradient of 12 degrees and a walking speed of 3.2 km/h [106]. This allows for an objective assessment of intermittent claudication severity and provides a starting point for ischemic preconditioning.

The role of walking training is to strengthen peripheral circulation, improve the blood supply to target organs, and improve the elasticity of arteries. Walking training, as the name suggests, involves walking at a constant pace, constant stride, and constant moderate speed. At the onset of pain that prevents walking, a break is taken until the pain subsides. Once the pain has subsided, the patient resumes walking at the same pace as before. Well-conducted walking training can increase intermittent claudication walking capacity by up to 180 m. Walking training should be carried out for at least 3 months, and the treadmill load should be intense enough to induce claudication, followed by a period of rest (Class of recommendation: I-A) [106].

If supervised walking training is not possible or not available, unsupervised exercise training/physical activity is recommended (Class I-C). In patients with intermittent claudication in whom the elimination of risk factors and the use of statins and walking training do not result in sufficient improvement, symptomatic pharmacotherapy may be considered to increase walking distance (IIb-A). If the patient’s normal functioning is impaired despite the implementation of walking training and conservative treatment, revascularization should be considered (IIa-C). If the patient’s normal functioning is significantly impaired, revascularization should be considered in combination with walking training and other methods (IIa-B).

However, the elderly often cannot perform walking training effectively or at all, for example, due to degenerative changes in the lower limb joints or post-stroke disability. In these cases, if the patient’s daily activities are significantly impaired, revascularization should be considered in combination with exercise that is feasible for the patient.

### 5.2. Head

Ischemia-adaptive methods, which involve enhancing hypoxia tolerance in patients with atherosclerosis of the carotid, vertebral, and cerebral arteries, are not as well established as walking training in PAD. However, due to the interesting results of several studies, they will be briefly presented here. Inducing a controlled ischemia in another arterial area, also known as remote ischemic conditioning (RIC), has been shown to improve cognitive function and harden the brain to hypoxia, both in primary prevention and following a stroke [188]. The test method is relatively simple and safe. A BP cuff is placed first on one arm and then on the other, inflated to 200 mmHg each time for about 5 min, then deflated for 5 min and inflated/deflated again 5 times for 5 min. The ischemia induced in the upper limb is designed to induce the body to activate adaptive mechanisms; improve motor rehabilitation after stroke; restore motor function in patients through remyelination, axonal regeneration, and stimulation of angiogenesis; and have antioxidant and protective effects against reperfusion brain damage [189]. Patients are currently being recruited for the trial (NCT05263531).

## 6. Closing Remarks and Future Directions

Atherosclerosis, vascular stiffness, and endothelial dysfunction accelerates with ageing [190,191,192]. Metabolic comorbidities, such as hypertension, diabetes mellitus, and dyslipidemia, are common risk factors for unfavorable vascular events. The evidence shows that early behavioral and medical intervention could halt early atherosclerotic changes, vascular stiffening, and remodeling, as well as restore endothelial function, provided that the best medical care is offered to the patient [193,194,195]. Conversely, young patient age causes serious delays in undertaking decisions on the treatment initiation. In this setting, some logistic and machine learning models could be used to time the initiation of the non-surgical interventions [55,196,197].

Despite this, in real-world settings, cardiovascular risk factors are undertreated and tend to accumulate with advancing age, along with multi-morbidity, cognitive decline, and increasing frailty [198,199]. In consequence, when atherosclerotic lesions cause severe arterial occlusive disease (frequently not limited to a single arterial territory), the surgical or percutaneous procedures are at remarkably increased risk, or do not bring the expected benefit. In particular, this is true for patients with type 2 diabetes, a metabolic disease resulting in pan-vascular complications that affects micro- and macro-vessels [200].

Furthermore, as in elderly patients, particularly those above age of 80, many co-existing morbidities present similar clinical symptoms. The overlapping of reporting symptom, e.g., involving musculoskeletal disorders and PAD, as well as cardiac and carotid morbidities, poses an important decision-making confounder [201,202]. It is worth mentioning that although atherosclerotic lesions affect numerous vascular territories, they aggressiveness, dynamics, and clinical presentation vary with ageing and between the territories. The age-associated low-grade chronic inflammation and associated extensive calcifications predispose to plaque rupture and erosion, and they are accelerators of plaque growth and vulnerability. This should be kept in mind, as for patients with chronic symptomatic PAD, smoking cessation and exercise training should be first-line management, as long as the patient can exercise. Conversely, in carotid disease, asymptomatic carotid stenosis should be monitored for signs of plaque instability. In addition, renovascular disease poses clinical dilemmas that cannot be easily resolved.

As we focus more on stabilizing atherosclerotic lesions and improving quality of life in the elderly and less on surgical and endovascular interventions for non-coronary artery stenosis, conservative management should be well planned in this patient population [203,204,205].

## Figures and Tables

**Table 1:** Comparison of atherosclerotic lesions in different arterial territories, with emphasis on diversities in the elderly patients.

Arterial Territory		Internal Carotid Arteries	Femoral Arteries	Coronary Arteries	Renal Arteries
Characteristics					
Risk factors	Main	Hypertension	Smoking, diabetes, age	Hyperlipidemia	Age, hypertension
	Others	Hyperlipidemia Age Diabetes Smoking	Hyperlipidemia Male gender	Diabetes Hypertension Male gender Age	Diabetes Hyperlipidemia Smoking
Predominant morphology and pattern of mature atherosclerotic plaque		Large lipid and necrotic core, decreasing thickness of fibrous cap, high infiltration of inflammatory cells, neo-angiogenesis, reduction in smooth muscle cells, propensity for plaque surface ulceration and embolization.	Few lipid and necrotic elements, thick fibrous cap, sparse vasa-vasorum, many smooth muscle cells, limited accumulation of inflammatory cells.	Large lipid and necrotic core, decreasing thickness of fibrous cap, high inflammatory cell infiltration, neo-angiogenesis, reduction in smooth muscle cells, plaque rupture leading to thrombosis and vessel occlusion.	Late manifestation of atherosclerosis, atherosclerotic lesions form at a later age and are therefore ‘young’ despite the older age of the individual. Plaque morphology: infiltration of inflammatory cells, lipid and necrotic core, excessive calcification process.
Diversities in the elderly patients		Severely calcified atherosclerotic lesions; stenosis in more than one carotid/vertebral artery; excessively elongated vessels, often with loop formation or acute angles <90%; high arterial stiffness and raised vascular resistance; atherosclerotic lesions in intracranial segments and cerebral arteries.	Diffuse, multilevel atherosclerotic lesions, also involving the tibiofemoral trunk and arteries below the knee; development of collateral circulation. In diabetics, superimposed lesions of diabetic angiopathy and peripheral neuropathy, hyalinization of the endothelium, ulcerations, trophic changes, reddening of the toes, and gangrene.	Stable atherosclerotic lesions, with a similar degree of progression and lumen reduction to those before age 70, but less prone to rupture and thrombosis. More fibrous components and calcification in plaques, fewer inflammatory cells and lower lipid composition, regression of necrotic and lipid core, better developed peripheral circulation.	Active atherosclerotic lesions, often coexisting with advanced atherosclerotic lesions in the coronary, carotid/vertebral arteries; abdominal aortic aneurysm and Leriche syndrome.
Typical clinical manifestation in the elderly	Often	Dizziness, memory impairment, tinnitus, progressive deterioration of cognitive functions, dementia, general disability, falls and their consequences, increasing frailty syndrome, stroke from large extracranial arteries.	Asymptomatic or mild intermittent claudication, whole-leg fatigue, numbness, leg pain on palpitation, hypoesthesia.	Dyspnea, easy fatigue, palpitations, atrial and ventricular arrhythmia chronic coronary syndrome, increasing symptoms of HF, reduced exercise tolerance.	Sudden worsening of blood pressure control, increasing symptoms of HF, decreased exercise tolerance, angina complaints, escalation of ACEI/ARB doses may cause acute renal failure, progression of of renal failure.
	Infrequently	Asymptomatic course.	Critical ischemia, acute lower limb ischemia, gangrene, non-healing ulcers, amputation.	Acute coronary syndrome with ST-segment elevation.	Pulmonary flash oedema, chronic coronary syndrome, acute renal failure.
Predominant mechanism of acute ischemia of the supplied organ	Often	Embolization of cerebral arteries, facilitated by the morphology and histopathological composition of the plaque and the distribution of stress shear forces resulting from the anatomy of the carotid artery bulb, 70–80%.	Decrease in blood flow, e.g., due to patient dehydration, infection, or calcification of the intimal layer. Obstruction of small peripheral arteries (favored by diabetes, dialysis).	Acute arterial occlusion due to plaque rupture and thrombus formation, 80–90%, mainly in men, often in women over the age of 60–65 years and above.	Hypertensive crisis, pulmonary oedema, acute circulatory failure.
	Rarely	Acute arterial occlusion due to plaque rupture and thrombus formation, 10–20%. Hypoperfusion mechanism, 5–10% (older people tend to have well-developed collateral cerebral circulation).	Non-atherosclerotic acute limb ischemia (e.g., cardiogenic embolism—thrombus, myxoma). Plaque rupture and thrombus (usually well-developed collateral circulation protects against acute lower limb ischemia).	Microvascular embolism—ulcerated plaques rich in proteoglycans, 10–20%, common etiology in women before the age of 60–65 years.	Acute renal ischemia—generally renal failure progresses slowly in a chronic manner—it is estimated that in about 20% of chronic dialysis patients, the cause is renal artery stenosis/obstruction.
Warning signs in the elderly		Syncope, loss of consciousness, dysregulation of previously well-controlled blood pressure. Other typical—transient limb weakness, amaurosis fugax, features of facial nerve palsy, hemiparesis.	Forced posture with the lower limb lowered (alleviates subjective symptoms of critical ischemia), inability to put the leg up (increases pain), swelling of the lower limb, ulceration, skin atrophy, redness and gangrene of the fingers.	Chest pain, sweating, pulmonary oedema, new-onset left bundle branch block on ECG, fatigue, nausea, abdominal pain. Chest pain is not always typical and characteristic	Dysregulation of previously well-controlled blood pressure, rapidly progressive renal failure, decreasing renal dimension on subsequent ultrasound examinations, 20–30% decline in eGFR after starting ACEIs, ARBs or SGLT2 inhibitors.
Methods of diagnosing atherosclerotic lesions in older people	Preferred	Doppler-duplex ultrasound—good resolution, no need for a contrast agent. Limitation—calcifications causing an acoustic shadow make it difficult to assess the vessel lumen. CT and MRI of the brain—diagnosis of ischemic lesions.	Doppler-duplex ultrasound—good resolution, no need for a contrast agent. Limitation—calcifications giving an acoustic shadow make it difficult to assess the vessel lumen. ABI indicates lower limb atherosclerosis when it is <0.9, but it is often nondiagnostic in people with diabetes due to intimal calcifications (ABI > 1.4). TBI in patients with diabetes and non-diagnostic ABI (>1.4); TBI < 0.7 is diagnostic for PAD. Pulse oximetry—measurement of blood pressure on the toe—a prognostic indicator of ulcer healing.	Treadmill test—assessment of exercise tolerance and detection of new ischemic changes. Echocardiography—assessment of left ventricular systolic function; wall motion abnormalities; diastolic function; exclusion of intracardiac problems such as thrombus, valve disease, pericardial effusion, features of pulmonary embolism. SPECT—assessment of viability and ischemic area on isotope.	Doppler-duplex ultrasound—good resolution, no need for a contrast agent. Limitation—obesity makes the examination difficult/impossible.
	Limited	CTA—anatomical assessment, but test affects renal and thyroid function, caution if renal failure with eGFR below 50 mL/min/kg, calcifications make assessment of vascular stenosis difficult. MRA—anatomical assessment, possible assessment of plaque morphology, but long examination, requires administration of gadolinium (caution if eGFR < 30 mL/min); claustrophobia.	CTA—anatomical assessment, but test affects renal and thyroid function; caution if renal failure with eGFR below 50 mL/min/kg, calcifications make assessment of vascular stenosis difficult. MRA—anatomical assessment; possible assessment of plaque morphology, but long examination, requires administration of gadolinium (caution if eGFR < 30 mL/min); claustrophobia.	CTA—anatomical assessment, but test affects renal and thyroid function; caution if renal failure with eGFR below 50 mL/min/kg; calcifications make assessment of vascular stenosis difficult. MRA—anatomical assessment; possible assessment of plaque morphology, but long examination, requires administration of gadolinium (caution if eGFR < 30 mL/min); claustrophobia.	CTA offers anatomical assessment, but it affects renal and thyroid function; caution if renal failure with eGFR below 50 mL/min/kg; calcifications make assessment of vascular stenosis difficult. MRA—anatomical assessment; possible assessment of plaque morphology, but long examination, requires administration of gadolinium (caution if eGFR < 30 mL/min); claustrophobia. Scintigraphy with captopril test—not recommended for diagnosis of RAS (class III recommendation). Renal vein renin activity assessment—not recommended (class III recommendations).

ABI—ankle-brachial index; CTA—computed tomography angiography; MRA—magnetic resonance angiography; RAS—renal artery stenosis; TBI—toe-brachial index.

## Data Availability

The data presented in this study are available on request from the corresponding author. The data are not publicly available due to privacy.
